# The *Chlamydia pneumoniae* Inclusion Membrane Protein Cpn1027 Interacts with Host Cell Wnt Signaling Pathway Regulator Cytoplasmic Activation/Proliferation-Associated Protein 2 (Caprin2)

**DOI:** 10.1371/journal.pone.0127909

**Published:** 2015-05-21

**Authors:** Rhonda Flores, Guangming Zhong

**Affiliations:** Department of Microbiology & Immunology, University of Texas Health Science Center at San Antonio, San Antonio, Texas, United States of America; University of California Merced, UNITED STATES

## Abstract

We previously identified hypothetical protein Cpn1027 as a novel inclusion membrane protein that is unique to *Chlamydia pneumoniae*. In the current study, using a yeast-two hybrid screen assay, we identified host cell cytoplasmic activation/proliferation-associated protein 2 (Caprin2) as an interacting partner of Cpn1027. The interaction was confirmed and mapped to the C-termini of both Cpn1027 and Caprin2 using co-immunoprecipitation and GST pull-down assays. A RFP-Caprin2 fusion protein was recruited to the chlamydial inclusion and so was the endogenous GSK3β, a critical component of the β-catenin destruction complex in the Wnt signaling pathway. Cpn1027 also co-precipitated GSK3β. Caprin2 is a key regulator of the Wnt signaling pathway by promoting the recruitment of the β-catenin destruction complex to the cytoplasmic membrane in the presence of Wnt signaling while GSK3β is required for priming β-catenin for degradation in the absence of Wnt signaling. The Cpn1027 interactions with Caprin2 and GSK3β may allow *C*. *pneumoniae* to actively sequester the β-catenin destruction complex so that β-catenin is maintained even in the absence of extracellular Wnt activation signals. The maintained β-catenin can trans-activate Wnt target genes including Bcl-2, which may contribute to the chlamydial antiapoptotic activity. We found that the *C*. *pneumoniae*-infected cells were more resistant to apoptosis induction and the anti-apoptotic activity was dependent on β-catenin. Thus, the current study suggests that the chlamydial inclusion protein Cpn1027 may be able to manipulate host Wnt signaling pathway for enhancing the chlamydial anti-apoptotic activity.

## Introduction

*Chlamydia pneumoniae*, a human respiratory pathogen, is associated with a spectrum of diseases such as atherosclerosis and asthma [1–4]. However, the precise mechanisms of *C*. *pneumoniae* pathogenicity remain unknown. All *Chlamydia* species, including *C*. *pneumoniae*, possess a unique obligate intravacuolar biphasic life cycle [5]. The developmental cycle begins with attachment and entry of an extracellular infectious elementary body (EB) to a host cell, most likely via an induced endocytosis [6]. An EB rapidly differentiates into a non-infectious but metabolically active reticulate body (RB). After multiple rounds of replication, the progeny RBs differentiate back into EBs for spreading to neighboring cells [7]. Throughout the chlamydial developmental cycle, chlamydial organisms remain within the cytosolic membrane-bound vacuole termed inclusion. The inclusion expands and is modified by the insertion of chlamydial inclusion membrane proteins (Incs). Incs have been proposed to play important roles in chlamydial exploitation and survival in the eukaryotic host [8–10]. Thus, extensive efforts have been made in identifying Incs [11–19]. As results, many chlamydial proteins have been localized in the inclusion membrane, including the hypothetical protein Cpn1027 [15]. Cpn1027 possesses the structural features common to most Incs, with a N-terminal bi-lobed hydrophobic region that may be localized in the membrane and a hydrophilic c-terminus that may be exposed to the host cell cytosol.

*Chlamydia* has evolved the ability to acquire nutrients and metabolic intermediates through the inclusion membrane [20,21]. Inc proteins are hypothesized to interact with host cell proteins during infection. The IncA protein was found to be required for homotypic vesicle fusion when a single cell is infected with multiple chlamydial organisms [8]. A *C*. *pneumoniae-*Inc protein designated Cp0236 was identified to interact with the host NF-ĸB activator 1 (Act1), thereby protecting *C*. *pneumoniae-*infected cells from interleukin 17-induced NF-ĸB activation [22]. The *C*. *pneumoniae* Inc protein Cpn0585 was found to interact with multiple Rab GTPases, which might contribute to the intracellular development of *C*. *pneumoniae* [9]. In addition, Chlamydia is known to possess a profound anti-apoptotic activity [23]. Although the chlamydial anti-apoptotic activity was mapped to the blockade of Bax/Bak activation [24,25], the precise mechanism remains unknown. It will be interesting to test whether any of the chlamydial Incs such as Cpn1027 can contribute to the chlamydial anti-apoptotic activity.

The canonical Wnt signaling pathway is evolutionarily conserved and it regulates crucial aspects of development and morphogenesis including cellular proliferation, differentiation, cell migration, and apoptosis partially through the accumulation and stabilization of β-catenin [26]. β-catenin has dual functions in mammalian cells: it is part of a complex of proteins that constitute adherens junctions and it is also a transcription factor, trans-activating Wnt target genes [26]. In the absence of Wnt signal, cytosolic β-catenin is degraded by a β-catenin destruction complex composed of Axin, adenomatosis polyposis coli (APC), glycogen synthase kinase 3β (GSK3β), and casein kinase 1α (CK1α). In this complex, β-catenin is phosphorylated by both GSK3β and CK1α, triggering the ubiquitination and subsequent degradation of β-catenin by the proteasome [26,27]. When Wnt signaling is activated by binding of Wnt ligands, which are usually secreted glycoproteins, to the 7-transmembrane protein Frizzled (Fz) and the single-pass low-density lipoprotein receptor-related protein 5/6 (LRP5/6) at the cell surface, LRP5/6 is phosphorylated to recruit the β-catenin destruction complex to the plasma membrane [26,27]. Cytoplasmic activation/proliferation-associated protein 2 (Caprin2) facilitates the phosphorylation of LRP5/6 by GSK3β and enhances the interaction between Axin and the cytoplasmic tail of LRP5/6, both of which are required for the stabilization of cytosolic β-catenin [28]. Interestingly, the β-catenin was previously found to recruit to the chlamydial inclusion in *C*. *trachomatis*-infected cells [29]. In normal cells, the subsequent rise in the cytoplasmic pool of β-catenin allows it to translocate into the nucleus, where it binds to the T-cell factor/lymphoid enhancer factor (TCF/LEF) family of transcription factors, displacing the co-repressor Groucho and histone deacetylases (HDAC), thus stimulating the transcription of Wnt responsive genes [26,27] as well as induce gene expression of anti-apoptotic proteins, such as Bcl-2 [30]. Therefore, β-catenin, regulated by GSK3β, is an important target for regulating apoptosis [31].

In the current study, we used the *C*. *pneumoniae* inclusion membrane protein Cpn1027 C-terminal region covering residues 210–527, designated as Cpn1027(210–527), as a bait to screen a HeLa cDNA library in a yeast two-hybrid assay and found that Cpn1027 interacted with host Caprin2, a regulator of the β-catenin destruction complex. Both a red fluorescence protein (RFP)-Caprin2 fusion protein and the endogenous GSK3β, a critical component of the β-catenin destruction complex, were recruited to the *C*. *pneumoniae* inclusion. The recruitment of the β-catenin destruction complex to the *C*. *pneumoniae* inclusion correlated with the *C*. *pneumoniae* anti-apoptotic activity and knocking down of β-catenin attenuated the *C*. *pneumoniae* anti-apoptotic activity.

## Materials and Methods

### 1. Yeast two-hybrid assay

For yeast two-hybrid screening, the DNA fragment coding for Cpn1027 C-terminal region covering residues 210–527 [designated as Cpn1027(210–527), Cpn1027 was also known as Cp0825 in the *C*. *pneumoniae* AR39 genome (http://www.stdgen.lanl.gov/)] was amplified from *C*. *pneumoniae* AR39 genomic DNA (forward primer, 5’-ACGCGTCGACATGGAACAAAATCTCTTTTTAAAA-3’; reverse primer, 5’- TTTTCCTTTTGCGGCCGCTTAAAGAGGTCCCTTAGGGAC-3’) and cloned into GAL4 DBD ProQuest two-hybrid bait vector pDBLeu, generating the plasmid pDBLeu-Cpn1027(210–527). The MaV203 yeast strain was transformed with pDBLeu-Cpn1027(210–527) as previously described [32] and the bait-expressing yeasts were made competent for screening a pre-made HeLa cell cDNA library (Invitrogen, Grand Island, NY 14072) constructed in pEXP-AD502 GAL4AD as prey. Yeast clones that permit interactions between bait and prey were selected on medium without Leu and Trp (-LW), or without Leu, Trp and His, plus 50mM 3-aminotriazole (3AT;-LWH 50mM 3AT) or without Leu, Trp and Uracil (-LWU). The library plasmids were extracted from the selected yeasts and processed for sequencing as described elsewhere [33]. One of the prey plasmids encoded the residues 245–1128 of host protein Caprin2, which was focused in the current study. A pair of positive control bait (pGal4DB-LMP1) and prey (pGal4AD-TRAF3) plasmids were kindly provided by Dr. Kenneth Izumi ([34].

### 2. Co-immunoprecipitation assay

HEK 293T or HeLa cells (both from ATCC, Manassas, VA, USA) were transfected or co-transfected with the following plasmids using the Lipofectamine 2000 transfection reagent following the protocol recommended by the manufacturer (Invitrogen). The pDsRed-Cpn1027, pDsRed-APC4 and pDsRed-Caprin2(245–1128) plasmids were created by cloning the genes of interest into the pDsRed Monomer C1 mammalian expression vector (BD Biosciences Clontech, San Jose, CA 95131), including full-length Cpn1027 covering residues 1–527 (forward, 5’-CGGGGTACCATGCCAGGTTCTGTGTCATC-3’; reverse, 5’- CGCGGATCCTTAAAGAGGTCCCTTAGGGAC-3’), APC4 (forward, 5’-CCGCTCGAGCTATGCCGTACCTTGGCTC-3’; reverse, 5’- CGGGGTACCTTATGATTTTATTTCTTCAATTGTTG-3’), and Caprin2 residues 245–1128 (forward, 5’-CCGGAATTCAATGCTCATACCAAATGACCAG-3’; reverse, 5’-CGGGGTACCTTAATCTTGATAAAGAAGATAGCC-3’). These genes were expressed as fusion proteins with a red fluorescent protein (RFP) tag fused to their N-termini. The Cpn1027 C-terminal region covering residues 210–527 (forward, 5’-CGGGGTACCATGGAACAAAATCTCTTTTTAAAA-3’; reverse, 5’-CGCGGATCCTTAAAGAGGTCCCTTAGGGAC-3’) was cloned into pFLAG-CMV-4 mammalian expression vector (Sigma, St Luis, MO) to create the recombinant plasmid pFLAG-Cpn1027(210–527). The Cpn1027(210–527) was expressed as a fusion protein with a FLAG tag fused to its N-terminus.

To confirm the interaction of Cpn1027 with Caprin2, HEK 293T cells were co-transfected with pFLAG- Cpn1027(210–527) and pDsRed-Caprin2(245–1128), pDsRed-APC4 or pDsRed alone. 24h after transfection, cells were lysed in immunoprecipitation buffer containing (20mM Tris-HCl, 130mM NaCl, 10% Glycerol, 1% Triton X-100, 2mM EDTA, 1 mM PMSF, 75 units aprotinin per ml, 20 mM leupeptin, and 200mM Na_3_VO_4_). The lysates were pre-cleared with protein G Sepharose beads (Amersham Biosciences, Waltham, MA). At the same time, the protein G Sepharose beads from Amersham Biosciences were incubated overnight at 4°C with rabbit anti-RFP polyclonal antibodies (raised with the RFP-GST fusion protein; unpublished data). Pre-cleared cell lysates were then incubated overnight at 4°C with the antibody bound Protein G beads. After incubation, Protein G-bound immunocomplexes were washed three times with PBS, resuspended in 2% SDS sample buffer, and subjected to Western blot analysis for protein-protein interactions.

To detect the interaction of Cpn1027 with GSK3β, HEK 293T cells were transfected with pDsRed-Cpn1027 or pDsRed alone. 24h after transfection, a mouse anti-GSK3α/β monoclonal antibody (BD Transduction Laboratories) was used to precipitate the cell lysates as described above. The precipitates were used for Western blot detection of RFP.

### 3. GST pull-down assay

The GST fusion proteins used for the GST pull-down assay were produced by cloning DNA coding for Cpn1027 or its fragments or Caprin2(245–1128) or its fragments into pGEX6p2 vector (Amersham Pharmacia Biotech) as described previously [35]. The primers used for the cloning were: Cpn1027 (forward, 5’-TCCCCCCGGGATGCCAGGTTCTGTGTCATC-3’; reverse, 5’-TTTTCCTTTTGCGGCCGCTTAAAGAGGTCCCTTAGGGAC-3’), Cpn1027 N-terminal region covering residues 1–209 or Cpn(1–209) (forward, 5’-TCCCCCCGGGATGCCAGGTTCTGTGTCATC-3’; reverse, 5’-TTTTCCTTTTGCGGCCGCTTAGATACATGCAATATCTTCTTT-3’), Cpn C-terminal region covering residues 210–527 or Cpn1027(210–527) (forward, 5’-CGCGGATCCATGGAACAAAATCTCTTTTTAAAA-3’; reverse, 5’- TTTTCCTTTTGCGGCCGCTTAAAGAGGTCCCTTAGGGAC -3’), Cpn1027(210–365) (forward, 5’- CGCGGATCCATGGAACAAAATCTCTTTTTAAAA-3’; reverse, 5’-TTTTCCTTTTGCGGCCGCTTATTCCTCAAAAAGTTGTACAAA-3’), and Cpn1027(366–527) (forward, 5’-CGCGGATCCATGCTCTGCCTAAAGCTTTTTA-3’; reverse, 5’- TTTTCCTTTTGCGGCCGCTTAAAGAGGTCCCTTAGGGAC-3’), Caprin2(245–1128) (forward, 5’-CCGGAATTCAATGCTCATACCAAATGACCAG-3’; reverse, 5’- TTTTCCTTTTGCGGCCGCTTATCTTGATAAAGAAGATAG-3’), Caprin2(245–905) (forward, 5’-CCGGAATTCAATGCTCATACCAAATGACCAG-3’; reverse, 5’- TTTTCCTTTTGCGGCCGCTTAGAACTGCAGCTGGCT-3’), and Caprin2(927–1128) (forward, 5’-CCGGAATTCATGGGAGGGACATCTGGT-3’; reverse, 5’- TTTTCCTTTTGCGGCCGCTTATCTTGATAAAGAAGATAG-3’). The Cpn1027 or its fragments were amplified using the *C*. *pneumoniae* AR39 genome as the template while Caprin2 or its fragments using the HeLa cell cDNA library (Invitrogen). Expression of the fusion proteins were induced with IPTG (Invitrogen) and the fusion proteins were extracted by lysing the bacteria via sonication in a Triton X-100 lysis buffer (1% Triton X-100, 1 mM PMSF, 75 units aprotinin per ml, 20 mM leupeptin, and 1.6 μM pepstatin). After high-speed centrifugation to remove debris, the fusion-protein containing supernatants were purified using agarose beads conjugated with glutathione (Amersham Biosciences) following the instructions provided by the manufacturer. After thorough washing, the fusion proteins immobilized onto beads were aliquoted and stored at -20°C. The amount of bead-bound fusion proteins was quantified based on Coomassie blue staining intensity on SDS-PAGE gel and the fusion proteins were adjusted to equivalent amounts for GST pull-down assays.

To validate Cpn1027 interaction with Caprin2, HEK 293T cells transfected with pDsRed-Cpn1027 or pDsRed alone for 24h were lysed using a lysis buffer containing 20mM Tris-HCl, 130mM NaCl, 10% Glycerol, 1% Triton X100, 2mM EDTA, 1 mM PMSF, 75 units aprotinin per ml, 20 mM leupeptin, and 200mM Na_3_VO_4_. The lysates were incubated overnight at 4°C with bead-immobilized GST-Caprin2(245–1128) beads. To map the interaction domains, HEK 293T cells transfected with pDsRed-Caprin2 for 24h were lysed as described above and the lysates were incubated overnight at 4°C with bead-immobilized GST-Cpn1027(1–209), GST-Cpn1027(210–527), GST-Cpn1027(210–365), GST-Cpn1027(366–527), or GST alone. To identify endogenous proteins, HeLa cell lysates were incubated overnight at 4°C with either bead-immobilized GST-Cpn1027 or GST alone. After incubation, beads were washed three times with PBS. Bound proteins were eluted using 2% SDS sample buffer and subjected to Western blot analyses.

### 4. Western blot assay

The Western blot assay was carried out as described elsewhere [36,37] for detecting components in the pellets brought down by agarose bead-immobilized protein G-antibody complexes (co-immunoprecipitation) or bead-immobilized GST fusion proteins (GST pull-down) or cultured cells. The pellets were boiled for 5 min in 2% sodium dodecyl sulfate (SDS) sample buffer while the cultured cells after various treatments including *C*. *pneumoniae* infection, siRNA transfection and apoptosis induction as described in sections below were lysed 2% SDS sample buffer and then boiled for 5 min. The boiled samples were resolved in SDS polyacrylamide gel and then transferred to nitrocellulose membranes for labeling with various primary and secondary antibodies. The primary antibodies used include the following: mouse polyclonal antibody against Cpn1027, rabbit polyclonal antibody against RFP/GST, goat polyclonal antibody against GST (GE Healthcare), mouse monoclonal antibody against β-actin (Sigma), mouse monoclonal antibody against GSK3α/β (BD laboratories), rabbit polyclonal antibody against Axin (Cell Signaling, Danvers, MA 01923), mouse monoclonal antibody against β-catenin (BD Laboratories), mouse monoclonal antibody against Bcl-2 (BD laboratories), mouse monoclonal antibody against chlamydial HSP60 (BC7.1), and rabbit polyclonal antibody against CK1α (Cell Signaling). The primary antibody binding was probed with a HRP (horseradish peroxidase)-conjugated secondary antibody and visualized with an enhanced chemiluminescence (ECL) kit (Santa Cruz Biotechnology). Semi-quantitative densitometry was performed using Bio-Rad Quantity One software.

### 5. ELISA

The interactions of GST fusion proteins with their binding partners were also measured by using a protein array enzyme-linked immunosorbent assay (ELISA) as described elsewhere [38,39]. The GST fusion proteins used for the ELISA were produced by cloning DNA coding for various Caprin2 fragments into pGEX6p2 vector (Amersham Pharmacia Biotech) as previously described [13]. The primers used for the cloning were: Caprin2(245–1128) (forward, 5’-CCGGAATTCAATGCTCATACCAAATGACCAG-3’; reverse, 5’- TTTTCCTTTTGCGGCCGCTTATCTTGATAAAGAAGATAG-3’), Caprin2(245–905) (forward, 5’-CCGGAATTCAATGCTCATACCAAATGACCAG-3’; reverse, 5’- TTTTCCTTTTGCGGCCGCTTAGAACTGCAGCTGGCT-3’), and Caprin2(927–1128) (forward, 5’-CCGGAATTCATGGGAGGGACATCTGGT-3’; reverse, 5’- TTTTCCTTTTGCGGCCGCTTATCTTGATAAAGAAGATAG-3’). Bacterial lysates containing the various GST fusion proteins or GST alone were added to 96-well microplates pre-coated with glutathione (Pierce, Rockford, IL). After washing and blocking, 1μg of purified Cpn1027(210–527) was added to each well of the microplates. A Cpn1027-sepciifc monoclonal antibody clone IE6 [15], was used to detect the protein-protein interactions and the antibody binding was detected with a goat anti-mouse IgG conjugated with HRP (Jackson ImmunoResearch Laboratories). The plate-immobilized HRP activity was quantitated using the soluble substrate 2,2’-azino-*bis*(3-ethylbenzothiazoline-6-sulfonic acid) diammonium salt (ABTS; Sigma) by reading the absorbance (optical density [OD]) at 405 nm using a microplate reader (Molecular Devices Corporation).

### 6. Immunofluorescence assay

HeLa cells grown on coverslips were fixed with 4% paraformaldehyde (Sigma) dissolved in PBS for 20 min at room temperature followed by permeabilization with 0.1% Triton X-100 (Sigma) for an additional 10 min. After washing and blocking, the cell samples were subjected to antibody and chemical staining. DAPI (blue; Sigma) was used to visualize nuclear DNA. A rabbit anti-chlamydial organism antibody (R12AR39, raised with *C*. *pneumoniae* AR39 organisms; unpublished data) or, in some cases, a rabbit polyclonal antibody against IncA (kindly provided by Dr. Ted Hackstadt, ref: [8], plus a goat anti-rabbit IgG secondary antibody conjugated with Cy2 (Jackson ImmunoResearch Laboratories) was used to visualize chlamydial inclusions. Mouse monoclonal antibodies raised against GSK3α/β (BD laboratories) or β-catenin (BD laboratories) plus a goat anti-mouse IgG conjugated with Cy3 (red, Jackson ImmunoResearch) were used to visualize the corresponding antigens. For the transfected cell samples, the RFP-fusion proteins were visualized via the fusion tag RFP (red). The images were acquired with an Olympus AX-70 fluorescence microscope equipped with multiple filter sets (Olympus) as described previously [36,37]. The single color images were superimposed with the software SimplePCI. All microscopic images were processed using Adobe Photoshop (Adobe Systems).

### 7. siRNA Knockdown and apoptosis induction

HeLa 229 cells were infected with *Chlamydia pneumoniae* AR39 at a multiplicity of infection of 0.5 in the presence of 2 μg/ml cycloheximide for different time points as indicated in individual experiments following an infection procedure as described elsewhere [15–18]. Twenty-4 hours after infection, the culture media were replaced with fresh media without cycloheximide. For knocking down β-catenin, HeLa cells were transfected with 100nM SignalSilence β-catenin-specific siRNA I (Cell Signaling) or with 100nM SignalSilence Control siRNA (Cell Signaling #6568) using the Lipofectamine 2000 following the protocol recommended by the manufacturer (Invitrogen). 24h after transfection, the cultures were either harvested for Western blot detection of the protein levels of β-catenin and Bcl-2 or induced to undergo apoptosis. For apoptosis induction, HeLa cells with or without *C*. *pneumoniae* or siRNA transfection were treated with 2μM staurosporine (STS) (Sigma) for 4 h. The mock treatment cells were incubated in culture medium alone. The cell samples were processed for immunofluorescence assay as described below. The cells fixed in 4% paraformaldehyde (Sigma) were processed for labeling both Chlamydia (green) and DNA (blue) as described above. Cells were evaluated for chromatin condensation [23,24,37] and the presence of either *C*. *pneumoniae* infection. For each sample, cells from ten random fields were counted under an Olympus AX-70 fluorescence microscope and the percentage of apoptotic cells was calculated as the number of apoptotic cells divided by the total number of cells counted, ~100 cells for each coverslip.

## Results

### 1. Cpn1027 interacts with host Caprin2

**Fig 1:**
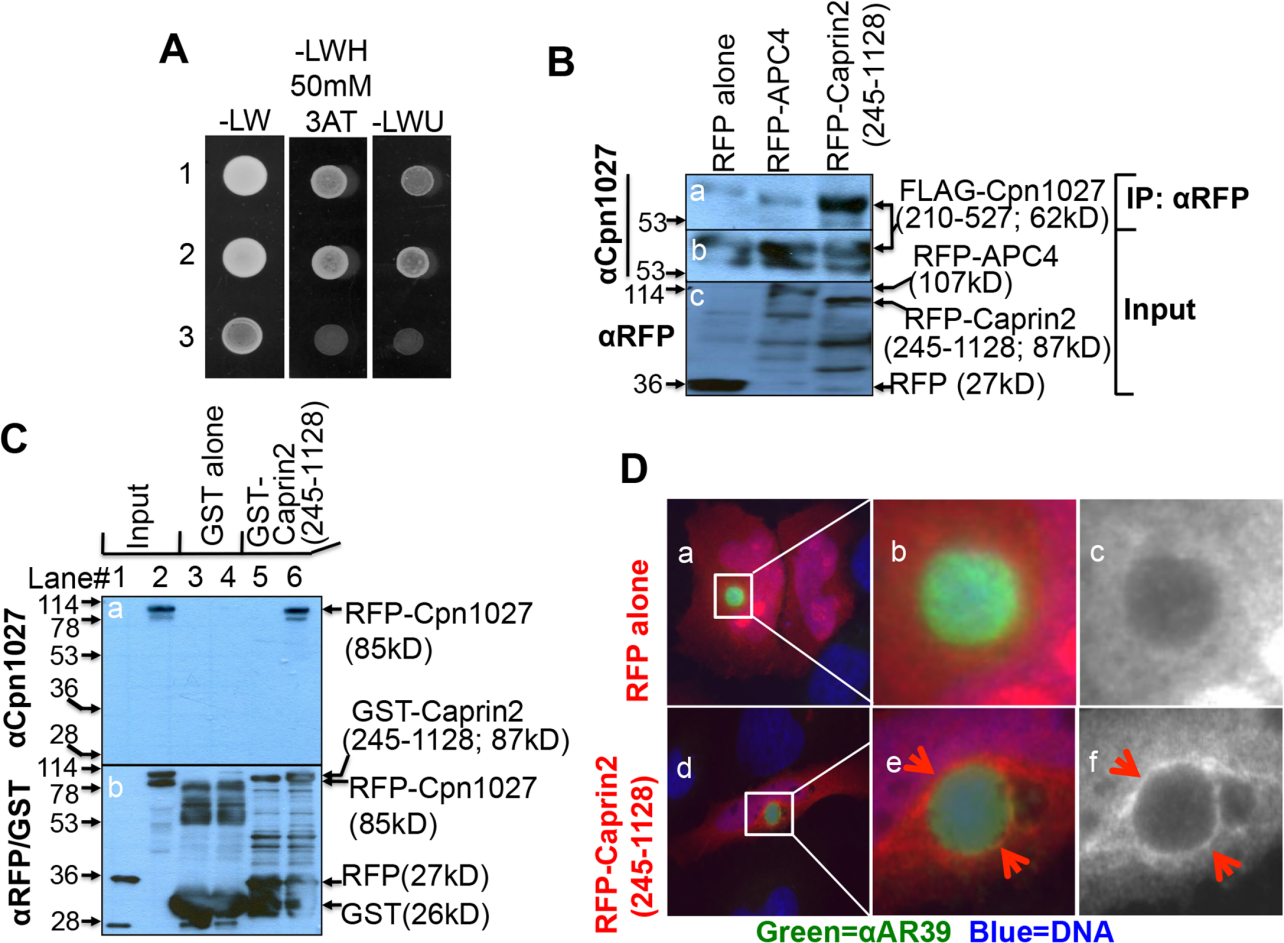
*C*. *pneumoniae* interaction with host protein Caprin2. (A) Yeasts co-transformed with pEXP-AD502-Caprin2(245–1128) as prey plus pDBLeu-Cpn1027(210–527) as bait (row 1) or the positive control prey pGal4AD-TRAF3 plus bait pGal4DB-LMP1 (row 2) or the negative control prey pGal4AD plus bait pGal4DB-LMP1 (row 3) were grown on plates with selective medium without Leu and Trp (-LW, left column) or without Leu, Trp and His but supplemented with 50mM *3-*Amino*-1*,*2*,*4-*triazole or 3AT (-LWH 50mM 3AT, middle column) or without Leu, Trp and Uracil (-LWU, right column). Note that bait-prey interactions occurred in rows 1 & 2 but not 3. (B) HEK 293T cells co-transfected with pFLAG-Cpn1027(210–527) and pDsRed vector (RFP alone, left lane), pDsRed-APC4 (RFP-APC4, middle lane) or pDsRed-Caprin2(245–1128) [RFP-Caprin2(245–1128), right lane] were harvested as whole cell lysates for immunoprecipitation using an anti-RFP antibody and the precipitates were probed with an anti-Cpn1027 antibody (panel a). The whole cell lysates were also directly probed with anti-Cpn1027 antibody (b) and anti-RFP antibody (c) respectively. Note that the anti-RFP antibody co-precipitated FLAG-Cpn1027(210–527) from cells co-transfected with pDsRed-Caprin2 but not pDsRed-APC4 or pDsRed vector alone. (C) Glutathione-S-transferase (GST) and GST-Caprin2(245–1128) fusion proteins were purified using glutathione-agarose beads as described in Materials and Methods. The GST (lanes 3 & 4) or GST-Caprin2(245–1128; lanes 5 & 6) beads were incubated overnight with lysates of HEK 293T cells transfected with pDsRed vector clone (expressing RFP tag alone, lanes 3 & 5) or pDsRed-Cpn1027 [expressing RFP-Cpn1027, lanes 4 & 6]. The bead-associated materials were resolved in SDS-PAGE for Western blot detection of Cpn1027 (a) and the tags RFP and GST (b). Note that the GST-Caprin2(245–1128) fusion protein but not GST alone pulled down RFP-Cpn1027. Samples loaded in lanes 1 & 2 were lysates without the GST pull down, thus marked as input on top of the figure. All GST or RFP tag-containing proteins were detected in panel b. (D) *C*. *pneumoniae* AR39-infected HeLa cells were transfected with pDsRed (expressing RFP alone, panels a-c) or pDsRed-Caprin2(245–1128) [expressing RFP-Caprin2(245–1128), d-f) as described in Materials and Methods. The chlamydial organisms were visualized with a rabbit anti-AR39 antiserum (αAR39 in green, stock# R12AR39) plus a Cy2-conjugated goat anti-rabbit IgG (green) and DNA with DAPI (blue). The areas marked with white squares in panels a & d were magnified and the magnified areas were shown in panels b (3 color-merged) & c (RFP alone in black and white) and e (3 color-merged) & f (RFP-Caprin2 in black and white) respectively. Note that the RFP-Caprin2 fusion protein but not RFP alone was detected around the inclusion membrane in the *C*. *pneumoniae* AR39*-*infected cells.

We used a C-terminal fragment (covering residues 210 to 527) of Cpn1027, designated as Cpn1027(210–527), as a bait to search for host cell binding partners from a HeLa cell cDNA library in a yeast two-hybrid screen assay. Our screen yielded several positive cDNA clones (data not shown) but only the clone encoding residues 245–1128 of Caprin2 was found to interact with Cpn1027 reproducibly in various *in vitro* assays (Fig 1). For example, when yeasts co-transformed with pEXP-AD502-Caprin2(245–1128) as prey plus pDBLeu-Cpn1027(210–527) as bait or the positive control prey Gal4AD-TRAF3 plus bait Gal4DB-LMP1 [34] or the negative control prey Gal4AD plus bait Gal4DB-LMP1 were grown on selective medium, the bait-prey interactions only occurred between Caprin2(245–1128) and Cpn1027(210–527) or TRAF3 and LMP1 but not Gal4AD alone and LMP1 (Fig 1A). In a co-immunoprecipitation assay, the RFP-Caprin2(245–1128) fusion protein but not the control RFP-APC4 fusion protein or RFP alone precipitated the FLAG-tagged Cpn1027(210–527) from the 293T cells co-transfected with plasmids coding for the RFP alone or RFP- and FLAG-tagged proteins (Fig 1B). The Cpn1027 interaction with Caprin2 was further confirmed in a GST pull down assay, in which the GST-Caprin2 (245–1128) but not GST alone pulled the RFP-Cpn1027 fusion protein but not RFP alone down into the pellets (Fig 1C). We also attempted to use Cpn1027 to immunoprecipitate endogenous Caprin2 from cell lysates of mock or *C*. *pneumoniae-*infected cells but without success. This might be primarily due to the fact that the endogenous Caprin2 was not detectable in cell lysates (data not shown). We also failed to precipitate the endogenous Cpn1027 from *C*. *pneumoniae*-infected cell lysates (data not shown), which has been a common challenge for probing the interactions of endogenous Incs with host cell proteins. Despite these technical difficulties, the 3 independent assays above have validated the interactions of *C*. *pneumoniae* Cpn1027 with host protein Caprin2. In addition, a RFP-Caprin2 (245–1128) fusion protein but not RFP alone was detected around *C*. *pneumoniae* inclusions (Fig 1D), suggesting that Caprin2 can be recruited by *C*. *pneumoniae* inclusion membrane proteins.

### 2. The C-terminal fragments of both Cpn1027 and Caprin2 are critical for the Cpn1027-Caprin2 interaction

**Fig 2:**
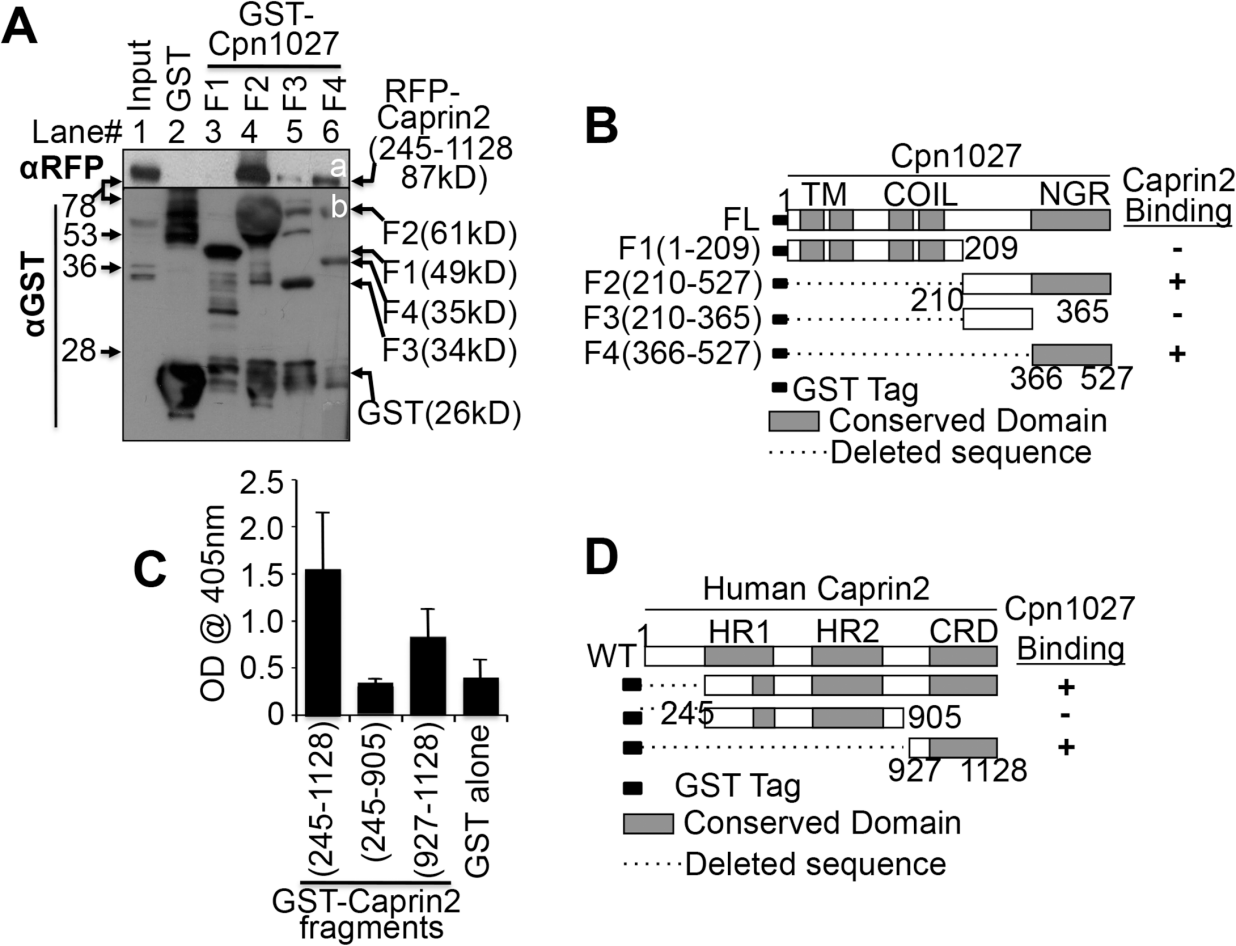
Identification of the Cpn1027-Caprin2 interaction domains. (A) Bead-immobilized GST (lane 2) or various GST-Cpn1027 fragments (from fragments 1 or F1, lane 3 to F2, lane 4, F3 lane 5 and F4, lane 6) as listed on the top of the panel were incubated overnight at 4°C with lysates made from HEK 293T cells transfected with pDsRed-Caprin2(245–1128) [expressing RFP-Caprin2(245–1128); The Caprin2(245–1128) fragment is the length of the cDNA clone identified during yeast two-hybrid screening). The precipitates pulled down by the beads were subjected to Western blot detection with anti-RFP (panel a) or anti-GST (b) antibodies. Note that GST-Cpn1027(366–527) or F4 was the shortest fragment that still pulled down a significant amount of RFP-Caprin2(245–1128). (B) Schematic representation of GST-Cpn1027 fragments (F1 to F4) and summary of their Caprin2 binding activities on the right side. Numbers indicate amino acid residue positions. The conserved domains include: TM, transmembrane domain; Coil, coil-coil domains; NGR, non-globular domain. The dashed line indicate the regions deleted from the corresponding constructs. (C) GST and GST-Caprin2 fragments as listed along the X-axis in the bottom of the figure were immobilized onto glutathione-coated microplates. 1μg of purified Cpn1027(210–527) protein (no GST tag, cleaved off from a GST fusion protein) was added to each well. The Cpn1027(210–527) bound to the plate-immobilized Caprin2 fragments was probed using an anti-Cpn1027 antibody followed by detection with a secondary goat anti-mouse IgG antibody conjugated with HRP plus a soluble substrate. The results were expressed as OD readings obtained at a wavelength of 405nm. Data from three independent experiments are shown. Note that GST-Caprin2 (927–1128) still maintained the ability to bind to Cpn1027. (D) Schematic representation of GST-Caprin2 fragments and summary of their Cpn1027 binding activities on the right side. WT Caprin2 depicts full-length Caprin2. Numbers indicate amino acid residue positions. The conserved domains include HR1, homologous region 1, HR2, homologous region 2 and CRD, C1q related domain.

Although we have shown that Cpn1027(210–527) interacts with Caprin2(245–1128), it remains unknown what shortest region of each is responsible for the interaction. Using a GST pull-down assay, we first mapped the interacting domain of Cpn1027(210–527). As shown in Fig 2A, both GST-Cpn1027(210–527) and GST-Cpn1027(366–527) but not GST-Cpn1027(210–365), GST-Cpn1027(1–209) or GST alone pulled down RFP-Caprin2(245–1128), suggesting that the Cpn1027(366–527) region is mainly responsible for interacting with Caprin2. We then mapped the region required for Caprin2 to interact with Cpn1027 in an ELISA assay (Fig 2C). The plate-immobilized GST-Caprin2(245–1128) and GST-Caprin2(927–1128) but not GST-Caprin2(245–905) or GST alone significantly bound Cpn1027(210–527), suggesting that the C-terminal fragment Caprin2(927–1128) is responsible for binding to Cpn1027.

### 3. Both Caprin2 and GSK3β, components of the β-catenin destruction complex, are recruited around the chlamydial inclusion membrane

**Fig 3:**
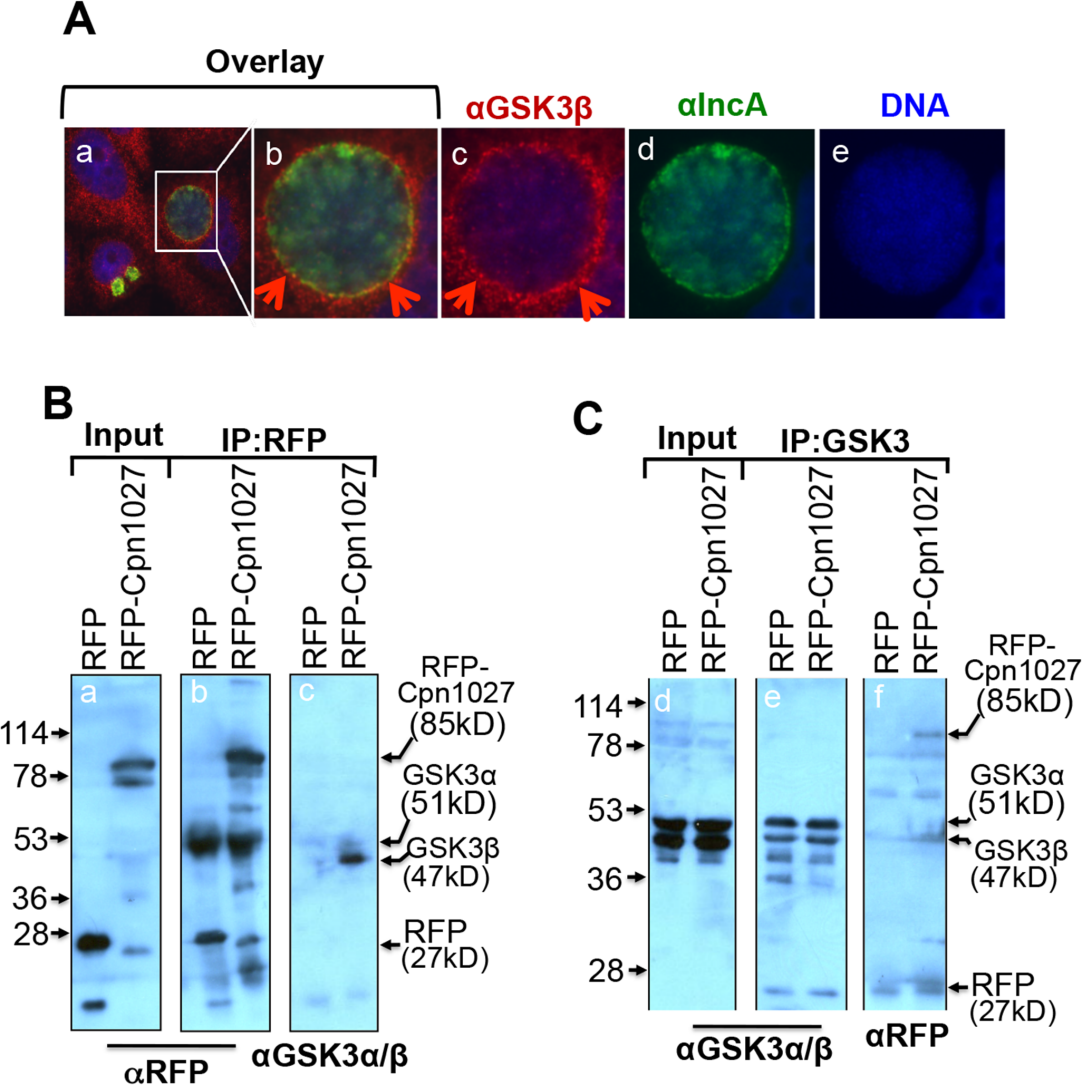
*C*. *pneumoniae* interaction with host GSK3β. (A) HeLa cells infected with *C*. *pneumoniae* AR39 organisms were processed for co-staining with a mouse anti-GSK3β plus a Cy3-conjugated goat anti-mouse IgG (red) and a rabbit anti-IncA antiserum plus a Cy2-conjugated goat anti-rabbit IgG (green). The DNA was labeled with DAPI (blue). Note that the endogenous GSK3β was detected around the chlamydial inclusions. The region marked with a white square in panel a was magnified and the magnified area was shown in panels b (3 color-overlay), c (GSK3β alone, red), d (IncA alone, green) and e (DNA alone, blue) respectively. (B) Lysates from HEK 293T cells transfected with pDsRed vector alone (expressing RFP alone, left lanes) or pDsRed-Cpn1027 plasmid [expressing RFP-Cpn1027, right lanes] were immunoprecipitated without (panel a) or with an anti-RFP antibody (b & c). The lysates or precipitates were analyzed by Western blot using an anti-RFP (panels a & b) or an anti-GSK3α/β (c) antibodies. Note that RFP-Cpn1027but not RFP alone co-precipitated the endogenous GSK3β. (C) The parallel lysates were precipitated without (panel d) or with an anti-GSK3α/β antibody (e & f). The lysate or precipitates were analyzed by using αGSK3α/β (panels d & e) or αRFP antibodies. Note that the endogenous GSK3β co-precipitated RFP-Cpn1027, but not RFP alone (f).

As Cpn1027 is an inclusion membrane-localized protein, interaction with Caprin2 should lead to the accumulation of Caprin2 in the vicinity of the chlamydial inclusion. We found that the ectopically expressed RFP-Caprin2 but not RFP alone was detected around the *C*. *pneumoniae* inclusion (Fig 1D), suggesting that Caprin2 is actively recruited to the *C*. *pneumoniae* inclusion membrane. In the presence of Wnt signaling, Caprin2 enhances the interaction of proteins in the β-catenin destruction complex with LRP5/6 to form a signalsome leading to the stabilization of cytosolic β-catenin [27]. In particular, Caprin2 promotes the GSK3β-mediated LRP5/6 interaction. Since Cpn1027 was found to interact with Caprin2, we next tested whether GSK3β was also recruited to the inclusion in *C*. *pneumoniae* infected cells. The endogenous GSK3β was detected at the periphery of the *C*. *pneumoniae* inclusion (Fig 3A). Furthermore, an anti-RFP antibody precipitated the endogenous GSK3β from HEK 293T cells expressing RFP-Cpn1027 but not RFP alone (Fig 3B). Vice versa, an anti-GSK3α/β antibody precipitated RFP-Cpn1027 but not RFP alone (Fig 3C). These observations together suggest that host cell GSK3β can be recruited around the *C*. *pneumoniae* inclusion by interacting with Cpn1027.

### 4. Knocking down β-catenin attenuate *C*. *pneumoniae* anti-apoptotic activity

**Fig 4:**
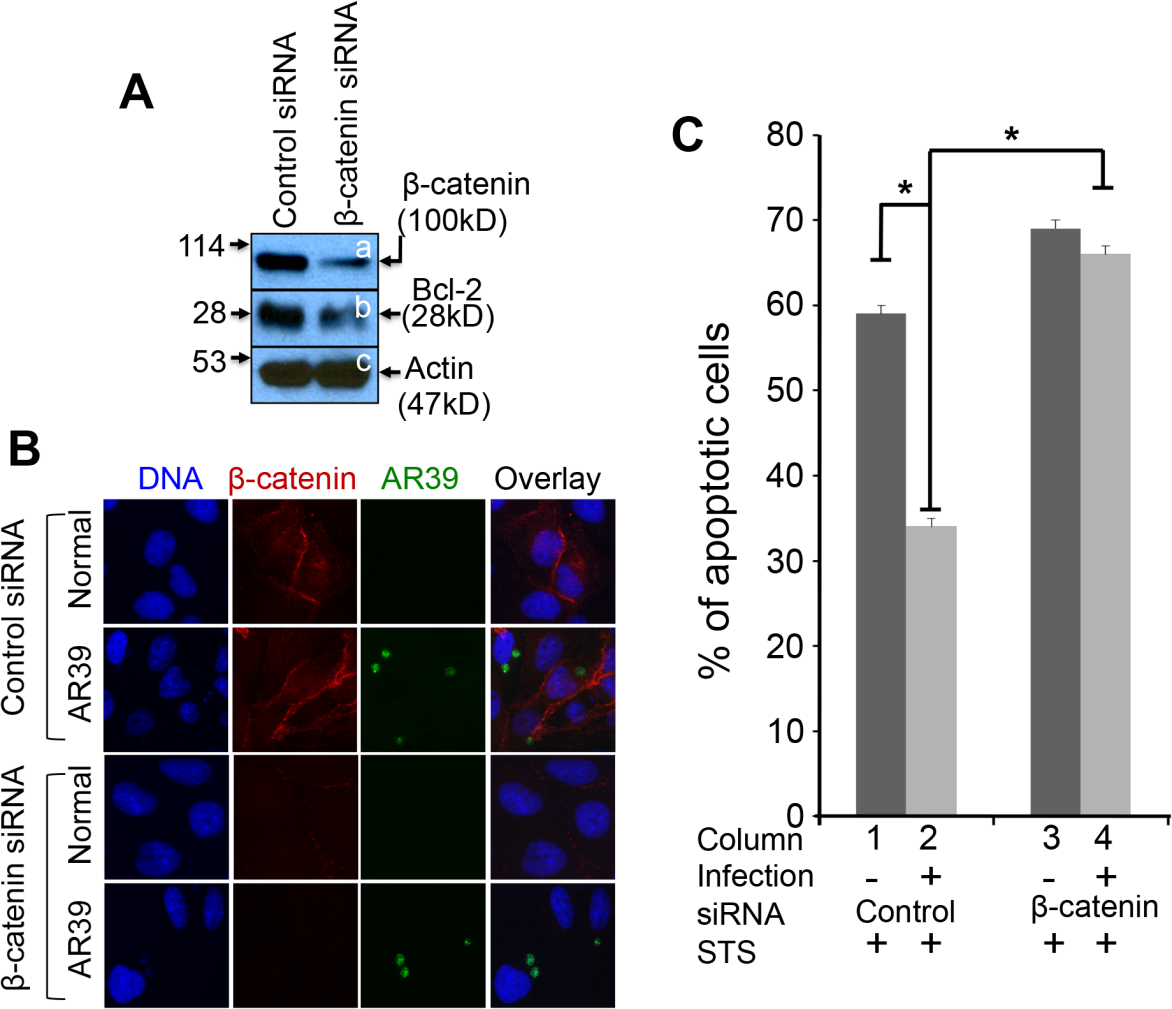
Effect of β-catenin knock-down on *C*. *pneumoniae* anti-apoptotic activity. HeLa cells infected with *C*. *pneumoniae* AR39 at a m.o.i. of 0.5 for 24h were transfected with control siRNA or β-catenin-targeted siRNA. (A) 24h after transfection, lysates were collected from a portion of the culture samples for detecting β-catenin (panel a), Bcl-2 (b) and β-actin (c) using the corresponding antibodies as listed in the materials and methods section. Note that cells transfected with β-catenin-targeted siRNA (right lane), but not control siRNA (left lane), displayed reduced levels of both β-catenin and Bcl-2. (B) The parallel cultures including the uninfected normal HeLa cells were also fixed for immunofluorescence assay for labeling *C*. *pneumoniae* AR39 (green), β-catenin (red) and DNA (blue) using the corresponding antibodies and dyes as listed in the materials and methods section. Note that the β-catenin SiRNA treatment significantly reduced the β-catenin protein level in both the AR-39-infected and normal uninfected cells. (C) The parallel cultures were further induced to undergo apoptosis using staurosporine (STS) stimulation for 4h. The apoptotic cells were identified using DNA staining. 10 random views were counted from each coverslip. Note that the *C*. *pneumoniae* AR39-infected cells were significantly resistant to apoptosis induction (column 1 v.s. 2) and the treatment with β-catenin-targeted siRNA but not control siRNA significantly neutralized the *C*. *pneumoniae* anti-apoptotic activity (column 2 v.s. column 4). Asterisks indicates significant differences of *P*<0.05; paired Student’s *t*-test. The data came from 3 independent experiments each with duplicates.

Since β-catenin plays a critical role in cell survival [26,28] and *C*. *pneumoniae* is known to possess a profound anti-apoptotic activity, we next tested whether knocking down β-catenin in *C*. *pneumoniae*-infected cells can inhibit the *C*. *pneumoniae* anti-apoptotic activity (Fig 4). HeLa cells with *C*. *pneumoniae* infection for 24h were transfected with either control siRNA or siRNA targeting β-catenin. 24h after transfection, a portion of the HeLa cell samples were harvested for monitoring the protein levels of β-catenin and Bcl-2. We found that the siRNA targeting β-catenin but not the control siRNA significantly reduced the levels of both β-catenin and Bcl-2 but not β-actin (Fig 4A). The parallel cultures including the uninfected normal HeLa cells were also fixed for immunofluorescence assay for monitoring the effect of the β-catenin SiRNA treatment (Fig 4B). The β-catenin protein level in both the AR-39-infected and normal uninfected cells was significantly reduced by the β-catenin SiRNA treatment. More importantly, some of the remaining cell culture samples were stimulated with staurosporine for inducing apoptosis and the percent of apoptotic cells was compared between the β-catenin-specific siRNA and control siRNA groups (Fig 4B). We found that in the presence of the control siRNA, *C*. *pneumoniae* maintained a robust anti-apoptotic activity. However, the siRNA that targets β-catenin blocked the *C*. *pneumoniae* anti-apoptotic activity.

## Discussion

In the current study, we have presented evidence that the *Chlamydia pneumoniae* inclusion membrane protein Cpn1027 can interact with the host Caprin2. First, in a yeast two-hybrid screening, the Caprin2-expressing clone was identified to be positive for interacting with Cpn1027, which was reproduced in a yeast co-transformation experiment. Second, the Cpn1027-Caprin2 interaction was validated in a co-immunoprecipitation assay, in which the RFP-Caprin2 fusion protein but not RFP fusion tag alone or RFP-APC4 fusion protein precipitated FLAG-Cpn1027. Third, a GST-Caprin2 fusion protein but not GST alone pulled down RFP-Cpn1027. Fourth, the interaction between Cpn1027 and Caprin2 was mapped to the C-termini of both molecules using a GST pull-down or ELISA assay. Finally, an RFP-Caprin2 fusion protein was recruited around *C*. *pneumoniae* inclusion membrane where Cpn1027 is localized. Since Caprin2 is a regulator in canonical Wnt signaling by facilitating the phosphorylation of the seven-transmembrane-spanning receptor Frizzled/LRP5/6 receptor complex and promoting the sequestration of the β-catenin destruction complex to the cytoplasmic membrane [28], the above observations suggest that *C*. *pneumoniae* may be able to recruit and sequestrate the β-catenin destruction complex around the inclusion membrane via the Cpn1027-Caprin2 interaction. This hypothesis is further supported by the finding that GSK3β, a key component of β-catenin destruction complex, was also recruited around the *C*. *pneumoniae* inclusion.

**Fig 5:**
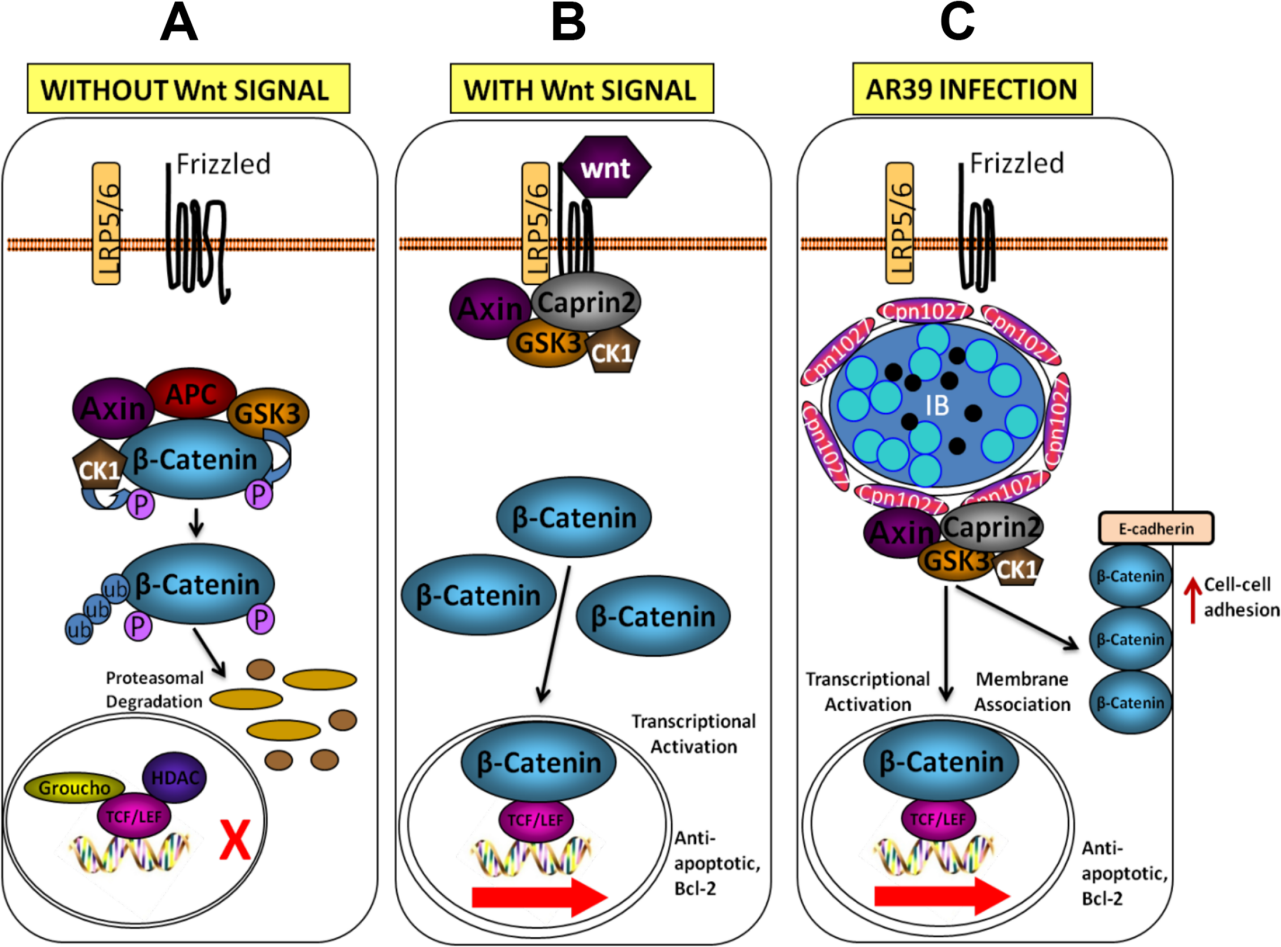
A working model for *C*. *pneumoniae*-mediated activation of the Wnt signaling transduction pathway. A. In the absence of Wnt signals, cytosolic β-catenin is degraded by a β-catenin destruction complex composed of Axin, adenomatosis polyposis coli (APC), glycogen synthase kinase 3β (GSK3β) and casein kinase 1α (CK1α). Both GSK3β and CK1α can phosphorylate β-catenin, which triggers ubiquitination and subsequent degradation of β-catenin by proteasomes. B. The Wnt signaling pathway is activated by binding of Wnt ligands, to Frizzled and LRP5/6 at the cell surface. LRP5/6 is activated by phosphorylation, which recruits the β-catenin destruction complex to the plasma membrane. The cytoplasmic activation/proliferation-associated protein 2 (Caprin2) facilitates LRP5/6 phosphorylation by GSK3β and enhances the interaction between Axin and the cytoplasmic tail of LRP5/6, which promotes the sequestration of the β-catenin destruction complex. The cytoplasmic pool of β-catenin rises and translocates into the nucleus, where it binds to the TCF/LEF family of transcription factors, displacing co-repressors Groucho and HDAC and acts as a co-activator to stimulate the transcription of anti-apoptotic, Bcl-2. C. In the *C*. *pneumoniae* AR39-infected cells, signalosomes are formed around the inclusion possibly through the chlamydial Inc protein Cpn1027 interacting with the β-catenin destruction complex, composed of Caprin2, Axin, GSK3β and CKIα resulting in the activation of the Wnt signaling transduction pathway in the absence of extracellular stimulation of Wnt. This leads to an increase in the cytoplasmic pool of β-catenin, nuclear β-catenin and membrane-associated β-catenin. The β-catenin-transactivated anti-apoptosis genes may promote the survival of the *C*. *pneumoniae* AR39-infected cells, hence the survival of AR39.

The observation that the C-terminal regions in both Cpn1027 and Caprin2 are responsible for their interactions indicates that their C-termini are accessible to each other. All chlamydial inclusion proteins possess a bi-lobed hydrophobic trans-membrane region in either their N- or C-terminal regions [19,40,41]. The Cpn1027’s bi-lobed region is localized in its N-terminus [15], suggesting that the C-terminus of Cpn1027 is exposed to the host cell cytoplasm, thus allowing the Cpn1027 C-terminus to access to the host cytoplasmic signaling protein Caprin2. It has been previously shown that the *Chlamydia pneumoniae* inclusion protein Cpn0585 may use its C-terminus to interact with multiple host cell Rab GTPases [9] while Cp0236 with the host NFkappaB activator 1 (Act1; ref: [22]. The C-terminus of Caprin2 is a C1q-related domain that is a homotrimer required for Caprin2 to interact with the Wnt signaling receptor complex LRP5/6 [28,42]. By interacting with the C1q-related domain of Caprin2, Cpn1027 anchored in the *Chlamydia pneumoniae* inclusion membrane may mimick the function of the Wnt signaling receptor for stabilizing the Caprin2 trimer and recruiting the β-catenin destruction complex around the chlamydial inclusion (Fig 5).

GSK3β, a key component of β-catenin destruction complex, was also localized around the *C*. *pneumoniae* inclusion. Furthermore, in cells expressing RFP-Cpn1027 (Fig 3) or GST-Cpn1027 (data not shown), the Cpn1027 fusion co-precipitated the endogenous GSK3β and the endogenous GSK3β also co-precipitated the Cpn1027 fusion protein. These observations further support our proposal that Cpn1027 can recruit the β-catenin destruction complex around chlamydial inclusions. However, at this moment, it remains unclear whether the interaction of Cpn1027 with GSK3β requires Caprin2. Since the endogenous Caprin2 is below our detection limit, we have not been able to determine whether the Cpn1027-GSK3β interaction is direct or indirect. Nevertheless, some chlamydial inclusion membrane proteins have been shown to interact with multiple host factors [9] and to simultaneously recruit multiple function-related host proteins around the inclusion [43]. Thus, regardless of the precise mechanisms on how the β-catenin destruction complex proteins are recruited around the *C*. *pneumoniae* inclusions, their close proximity to chlamydial inclusions may be biologically significant.

Caprin2 refers to cytoplasmic activation/proliferation-associated protein 2, which is normally distributed in the host cell cytosol. In response to the activation of Wnt signaling pathway, Caprin2 is recruited to the plasma membrane where it participates in the LRP5/6 signalosome formation by enhancing the interactions between Axin, GSK3β, and LRP5/6, resulting in the sequestration of the β-catenin destruction complex, inactivation of GSK3β, accumulation of β-catenin and translocation of β-catenin to the nucleus for trans-activating Wnt target genes [26–28]. On the contrary, in the absence of Wnt ligands, GSK3β in concert with other β-catenin destruction complex components Axin, APC, and CK1α, phosphorylates β-catenin, resulting in destruction of β-catenin. Cytoplasmic β-catenin levels are normally kept low through continuous proteasome-mediated degradation regulated by the β-catenin destruction complex. We propose that in *Chlamydia pneumoniae*-infected cells, Cpn1027 anchored in the inclusion membrane protein may mimick the function of the Wnt signaling receptor LRP5/6 by interacting with Caprin2 and recruiting β-catenin destruction complex around chlamydial inclusion, resulting in β-catenin accumulation and activation of Wnt signaling pathway-targeted genes as proposed in Fig 5. Thus, even in the absence of extracellular Wnt signaling, Cpn1027 may be able to deliver the Wnt-like signaling from inside cells.

The Chlamydia-triggered Wnt-like signaling may have multiple effects on the infected cells. In the current study, we found that the *C*. *pneumoniae*-infected cells were more resistant to apoptosis induction by staurosporine. β-catenin seemed to play an important role in the *C*. *pneumoniae* anti-apoptotic activity since knockdown of β-catenin significantly attenuated the *C*. *pneumoniae* anti-apoptotic activity. Thus, we proposed that preserving of β-catenin by sequestrating the β-catenin destruction complex around the chlamydial inclusions might represent an active mechanism for maintaining the *C*. *pneumoniae* anti-apoptotic activity. We are aware that inhibition of host cell apoptosis may involve multiple molecular mechanisms since maintaining host cell viability is fundamental for the slow growing *C*. *pneumoniae* organisms to complete their growth cycles. Since Cpn1027 is unique to *C*. *pneumoniae*, it should be cautious when applying the current finding to *C*. *trachomatis*. In *C*. *trachomatis*-infected cells, the β-catenin was found to recruit to the chlamydial inclusion [29], which may suppress the trans-activation activity of β-catenin. Nevertheless, the current study has provided the first experimental demonstration for the involvement of an inclusion membrane protein in manipulating the Wnt signaling pathway and promoting the *C*. *pneumoniae* anti-apoptotic activity.
